# Adipose-Derived Stem Cell Features and MCF-7

**DOI:** 10.3390/cells10071754

**Published:** 2021-07-11

**Authors:** Giuseppe Garroni, Francesca Balzano, Sara Cruciani, Renzo Pala, Donatella Coradduzza, Emanuela Azara, Emanuela Bellu, Maria Laura Cossu, Giorgio C. Ginesu, Ciriaco Carru, Carlo Ventura, Margherita Maioli

**Affiliations:** 1 Department of Biomedical Sciences, University of Sassari, Viale San Pietro 43/B, 07100 Sassari, Italy; giugarroni21@gmail.com (G.G.); mariafrancesca22@virgilio.it (F.B.); sara.cruciani@outlook.com (S.C.); renzopala6@gmail.com (R.P.); donatella.coradduzza@libero.it (D.C.); ema.bellu@hotmail.it (E.B.); carru@uniss.it (C.C.); 2 Institute of Biomolecular Chemistry, National Research Council, 07100 Sassari, Italy; emanuelagigliola.azara@cnr.it; 3 Department of Medical, Surgical and Experimental Sciences, General Surgery Unit 2 “Clinica Chirurgica”, University of Sassari, Viale San Pietro 8, 07100 Sassari, Italy; mlcossu@uniss.it (M.L.C.); ginesugc@uniss.it (G.C.G.); 4 Laboratory of Molecular Biology and Stem Cell Engineering, National Institute of Biostructures and Biosystems-Eldor Lab, Innovation Accelerator, Consiglio Nazionale Delle Ricerche, 40129 Bologna, Italy; ventura.vid@gmail.com; 5 Center for Developmental Biology and Reprogramming (CEDEBIOR), Department of Biomedical Sciences, University of Sassari, Viale San Pietro 43/B, 07100 Sassari, Italy

**Keywords:** adipose-derived stem cells, cellular mechanisms, epigenetics, cell proliferation, stemness genes, autophagy

## Abstract

Human adipose tissue-derived stem cells (hADSCs) are highly suitable for regeneration therapies being easily collected and propagated in vitro. The effects of different external factors and culturing conditions are able to affect hADSC proliferation, senescence, differentiation, and migration, even at the molecular level. In the present paper, we exposed hADSCs to an exhausted medium from the breast cancer cell line (MCF-7) to evaluate whether the soluble factors released by these cells may be able to induce changes in stem cell behavior. In particular, we investigated the expression of stemness-related genes (OCT4; Sox 2; Nanog), the cell-cycle regulators p21 (WAF1/CIP1) p53, epigenetic markers (DNMT1 and Sirt1), and autophagy-related proteins. From our results, we can infer that the exhausted medium from MCF-7 is able to influence the hADSCs behavior increasing the expression of stemness-related genes, cell proliferation, and autophagy. Polyamines detectable in MCF-7 exhausted medium could be related to the higher proliferation capability observed in hADSCs, suggesting direct crosstalk between these molecules and the observed changes in stem cell potency.

## 1. Introduction

Stem cells have been largely explored in the branch of regenerative medicine, being able to replace damaged elements for their ability to undergo a differentiation process upon request [1]. They are a suitable tool for regeneration therapies due to their simple isolation procedure and high proliferative capability in culture [2], low immunogenicity and immunosuppressive properties, being more stable in long-term culture, with lower senescence and greater proliferation capabilities as compared to other kinds of stem cells [3,4].

Human adipose-derived stem cells (hADSCs) are able to differentiate into different cell lineages upon stimulation using a conditioned medium, as demonstrated in several studies [5,6,7].

In particular, small molecules or physical energies from the microenvironment can act by modulating and modifying different pathways, such as stem cell self-renewal, inducing differentiation, and cell reprogramming [8,9,10].

This kind of behavior depends on stemness genes, including the octamer-binding transcription factor 4 (OCT4), SOX2, and Nanog [11], involved and expressed in undifferentiated stem cells [12], being related not only to the maintenance of an undifferentiated state but also to cell proliferation [13].

OCT4 is a key marker of stemness, implicated in lineage specification and reprogramming of somatic cells in vitro, together with SOX2 [14,15].

NANOG is a stem cell transcription factor playing an important role in the regulation of human development; it is involved in cell fate determination, proliferation, and apoptosis [16,17].

OCT4, Nanog, and SOX2 dysregulation has been related to different kinds of tumors, being upregulated in breast, colorectal, and liver cancers [18,19,20].

Together with stemness modulation, epigenetic modifications play an important role in stemness maintenance, cell differentiation, and the development of neoplasia [21,22,23]. Sirtuin 1 (Sirt1) and Dnmt1 DNA (cytosine-5)-methyltransferase 1 (DNMT1) affect both cell proliferation and differentiation, being associated with several cancer processes, and regulating cell cycle-associated genes [24,25,26,27].

These observations highlight the relevance of the stem cell microenvironment in conditioning fate decisions and adaptation processes. hADSCs have been widely used in regenerative and aesthetic/plastic reconstruction medicine being transplanted with different modalities in recipient injured tissues, whose derangement was not depending upon pre-existing malignancies, but it was rather resulting from hypoxic/ischemic, traumatic, and inflammatory conditions.

Nevertheless, autologous hADSCs and adipose tissue derivatives are increasingly used after mastectomy for breast cancer removal [28], with the aim of developing new approaches to breast reconstruction and tissue augmentation with natural appearance and texture. Under these clinical and surgical circumstances, it is conceivable that the recipient tissue is still retaining, at least in part, the molecular cues of a tumor microenvironment, as it has been consistently shown in the case of cancer cell dormancy and reentry even after thorough surgery and chemo-/radio-therapy [29]. The tumor microenvironment (TME) is a complex system comprising different cell populations as adipocytes, fibroblasts, and different stem and progenitor cells [30,31], interacting with soluble secreted factors in tumor progression [32]. The same microenvironment also encompasses different organic compounds capable of modulating cellular decisions at different interconnected levels. Among these molecules, polyamines are able to influence cell behavior in different situations. Their interaction with nucleic acids, proteins, and negatively charged phospholipids results in a change in their structure and conformation [33,34]. Thus, polyamines regulate cell growth and proliferation, as well as stem cell differentiation [35]. They are also involved in necrosis, apoptosis, and autophagy. Consonant with their pleiotropic features, polyamine levels are often dysregulated in cellular aging, cancer, and other hyperproliferative diseases [36].

We have previously highlighted the relevance of the tumor microenvironment in stem cell dynamics, showing that an exhausted medium from human hepatocarcinoma cell line HepG2 was able to remarkably affect both the stemness and the proliferative state in Wharton jelly mesenchymal stem cells [37].

In the present study, we investigated whether an exhausted medium from the breast cancer cell line MCF-7 may alter a number of essential features in hADSCs, including stemness, proliferation, and autophagy. We also assessed whether the same exhausted medium may prime an hADSC switch toward a malignant phenotype.

In particular, we analyzed the expression of the stemness-related genes, NANOG, OCT4, SOX2, the epigenetic modulator genes DNMT1, SIRT1, and the expression of p53 and its ability to modulate cell-cycle arrest/progression by p21 (WAF1/CIP1), and apoptosis induction by BAX. Here, we also decided to assess the polyamine content in MCF-7-exhausted medium and in the course of autophagy, a fundamental cell survival process through which cells are able to adapt to metabolic stresses. In particular, autophagy appears to contribute to the survival of cancer (stem)cells, which are forced to a high requirement for nutrients and oxygen to ensure their proliferation [38]. Moreover, cell fate and stem cell quiescence, activation, differentiation, self-renewal, and proliferation are influenced by autophagy [39].

## 2. Materials and Methods

### 2.1. Adipose-Derived Stem Cell Isolation and Culturing

Human Adipose-derived Stem cells (hADSCs) were obtained from subcutaneous adipose tissue of human adult patients during surgery procedures (*n* = 6, age = 45 ± 15 years, BMI: 22 ± 3 kg/m^2^), as previously described in Basoli V. et al. (2016) [5].

The study was approved by the Ethics Committee Review Boards for Human Studies in Sassari (n_ ETIC 240I/CE 26 July 2016, Ethical committee, ASL Sassari). All patients signed written informed consent. Before isolation, the fat tissue was washed two times with sterile Dulbecco’s phosphate-buffered saline (DPBS) (Euroclone, Milano, Italy) with 200 U/mL penicillin—0.1 mg/mL streptomycin (Euroclone, Milano, Italy). Tissue biopsies were mechanically reduced to small fragments by sterile scalpels and enzymatically digested in a solution of Hanks’ salts, CaCl_2,_ and 0.1% Type I Collagenase (Gibco Life Technologies, Grand Island, NY, USA) at 37 °C for 1 h to separate hADSCs from the stromal vascular fraction (SVF), and from mature adipocytes, that were removed. After the incubation time, the samples were filtered in a 70 μm cell strainer (Euroclone, Milano, Italy) and centrifuged 10 min at 600× *g* to separate the different cell fractions. The resulting pellets of SVF were resuspended into a hADSCs growth medium with basic Dulbecco’s Modified Eagle’s Medium (DMEM) (Life Technologies Grand Island, NY, USA) supplemented with 20% fetal bovine serum (FBS) (Life Technologies, Grand Island, NY, USA), 200 mM L-glutamine (Euroclone, Italy), and 200 U/mL penicillin—0.1 mg/mL streptomycin (Euroclone, Milano, Italy) and transferred in an incubator at 37 °C and 5% CO_2_. The culture medium was replaced every 3 days. hADSCs at confluence were detached from the flask using 0.25% EDTA trypsin (Euroclone, Milan, Italy), counted, and then immunomagnetically separated from the SVF and characterized by flow cytometry [5]. Flow cytometry analysis was exploited to assess the percentage of mesenchymal markers and the homogeneity of the isolated population. After fixation, cells were permeabilized and incubated with primary antibodies directed against CD73, CD90 (BD Biosciences, San Jose, CA, USA), CD105 (Santa Cruz Biotechnology, Heidelberg, Germany), CD45, and CD31 (Sigma-Aldrich, Munich, Germany) (all at 1 µg/106 cells) for 1 h at 4 °C, and with 1 µg of fluorescein isothiocyanate (FITC)-conjugated secondary antibody for 1 h at 4 °C in the dark. After washing, cells were analyzed on a flow cytometer (CytoFlex, Backman Coulter, Milan, Italy) as previously described in Basoli V. et al. (2016) [5].

### 2.2. MCF-7 Cell Culturing

MCF-7 cell line (ATCC, Manassas, VA, USA) were maintained in Dulbecco’s Modified Eagle’s Medium (DMEM) (Life Technologies Grand Island, NY, USA) supplemented with 10% fetal bovine serum (FBS) (Life Technologies, Grand Island, NY, USA), 200 mM L-glutamine (Euroclone, Italy), and 200 U/mL penicillin—0.1 mg/mL streptomycin (Euroclone, Milano, Italy). Cells were grown in 75 cm^2^ tissue culture flasks in the culture incubator at 37 °C with 5% CO_2_ and saturated humidity. The exhausted medium was collected, after 4, 7, and 10 days in culture, when the cells reached 80%–90% confluence, then it was centrifuged for 5 min at 600× *g* and filtered in a 0.22-μm cell strainer (Primo^®^ Syringe Filters Euroclone, Milano, Italy). From here on, the exhausted medium obtained from MCF-7 (MCF-7-EM) cultured for 4, 7, or 10 days will be referred to as 4d-MCF-7-EM, 7d-MCF-7-EM, and 10d-MCF-7-EM, respectively.

### 2.3. ADSC Treatment

**Figure 1:**
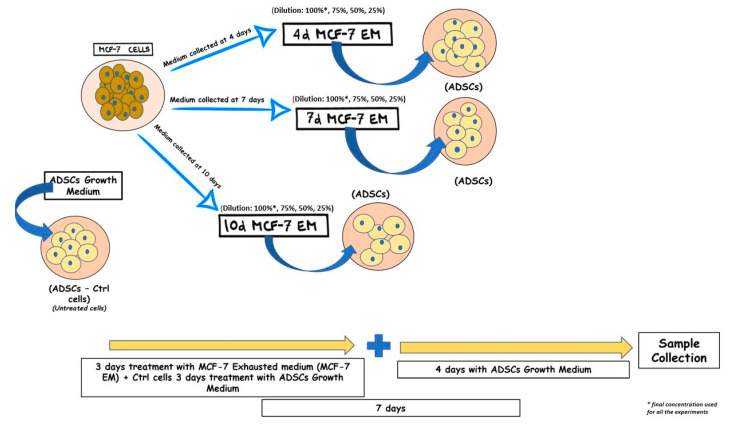
MCF-7 cell culturing and hADSC treatment.

hADSCs were seeded on 6-well plates and then exposed for 72 h to 1.5 mL of 4d-MCF-7-EM, 7d-MCF-7-EM, or 10d-MCF-7-EM. A group of cells was exposed to 1.5 mL of hADSCs basic growth medium, representing the cells used as untreated controls in the study, indicated as Ctrl. The 6-well cell culture plates were placed in a 37 °C incubator with 5% CO_2_ and saturated humidity. After the 3 days of treatment, the medium was removed and replaced by 1.5 mL of fresh hADSCs basic growth medium for an additional 4 days before proceeding with the following analysis, for a total incubation time of 7 days (Figure 1).

### 2.4. BrdU Cell Proliferation Assay

For the BrdU (5-Bromo-2-Deoxyuridine) proliferation assay, cells were seeded on 24-well plates and treated with MCF-7-EM at different time points. A group of cells was exposed to hADSCs basic growth medium, representing the cells used as untreated controls in the study, indicated as Ctrl. The proliferation assay BrdU Cell Proliferation Assay Kit (Cell Signaling Technology, Danvers, MA, USA) was performed. According to the manufacturer’s protocol, cells were fixed and incubated with 1X detection antibody solution and then with 1X HRP-conjugated secondary antibody solution. At the end of the incubation time, absorbance was monitored by spectrophotometric reading at 450 nm (Akribis Scientific, Common Farm, Frog Ln, Knutsford WA16 0JG, Great Britain).

### 2.5. MTT Viability Assay

hADSCs were exposed to different concentrations (100%, 75%, 50%, 25%) of the MCF-7-exhausted medium collected after 4, 7, and 10 days to evaluate its possible cytotoxic effect.

The colorimetric test of MTT 3-(4,5-Dimethylthiazol-2-yl)-2,5-Diphenyltetrazolium Bromide) (Sigma-Aldrich, Saint Louis, MO, USA) was used. Vital cells were able to reduce this compound, producing formazan that can be quantified by spectrophotometer at 570 nm. The hADSCs were seeded at a concentration of 5000 cells/well in 96-well plates. Cells used as untreated control were grown in the only basic growing medium. At the end of the incubation period, the medium containing the extracts was removed, and 100 µL of MTT at the final concentration of 0.65 mg/mL was added to each well and incubated for 2 h. After incubation, the formazan was dissolved in DMSO, and the absorbance was detected by spectrophotometric reading at 570 nm (Akribis Scientific, Common Farm, Frog Ln, Knutsford WA16 0JG, Great Britain). The viability of cultured cells in the presence of exhausted medium was calculated as % cell viability compared to the untreated control:

(OD570 treated cells) × 100/(OD570 control) considered as 100)

### 2.6. RNA Extraction and Real-Time Quantitative Polymerase Chain

Total mRNA was isolated from hADSCs cultured in the above-described conditions using PureLinkTM RNA Mini kit (Ambion, Life Technologies), according to the manufacturer’s protocol. Quantity and purity of RNA were measured using Nanodrop (Thermo Scientific, Waltham, MA, USA). Quantitative polymerase chain reaction (RT- qPCR) was performed using a CFX Thermal Cycler (Bio-Rad) in triplicate under standard RT-- qPCR conditions (55 °C for 10 min, 95 °C for 1 min, and then cycled at 95 °C for 10 s, 60 °C for 30 s, for 40–45 cycles), using Luna^®^ Universal One-Step RT-qPCR Kit (New England Biolabs). Luna Universal One-Step Reaction Mix (2X) 10 µL, Luna WarmStart^®^ RT Enzyme Mix (20X) 1 µL, 0.4 µM (0,8 µL) of each primer, and 2.5 µL (2.5 ng) of the total RNA template were mixed in 20 µL volumes and added to each reaction. Relative expression was determined using the “delta-CT method” with Glyceraldehyde-3-Phosphate Dehydrogenase (GAPDH) as a reference gene. The mRNA levels of treated cells were expressed as fold of change (2^−∆∆Ct^) of mRNA levels observed in control untreated cells. The RT-qPCR analysis was performed for the following set of genes: NANOG, Oct-4, SOX2, p53, p21 (WAF1/CIP1), Sirt1, and DNMT1. All primers used (Thermo Scientific, Waltham, MA, USA) are described in Table 1.

### 2.7. Autophagosome Detection Assays

To investigate the accumulation of autophagosomes in hADSCs cultured under the above-described conditions, Autophagosome Marker Antibody Sampler Kit (Cell Signaling Technology, Danvers, MA, USA) was used. After 7 days in culture, hADSCs were lysed by adding 1X SDS sample buffer according to the manufacturer’s protocol. Samples were then denatured at 95–100 °C for 5 min and loaded onto an SDS-PAGE gel. After transferring to a nitrocellulose membrane, samples were incubated in blocking buffer for 1 h at RT (room temperature) and then in primary antibody ON (Overnight) at 4 °C. At the end of incubation, the membrane was washed three times in TBS and incubated with an HRP-conjugated secondary antibody for 1 h at RT. Protein expression was assessed by SuperSignal Chemiluminescent HRP Substrates (Thermo Fisher Scientific, Grand Island, NY, USA).

### 2.8. Polyamine Analysis

#### 2.8.1. Chemicals

The reference standards of putrescine, cadaverine hydrochloride, spermidine hydrochloride, spermine, agmatine sulfate salt, N-acetyl-putrescine hydrochloride, N-acetyl-spermine trihydrochloride, N-acetyl-spermidine dihydrochloride, L-ornithine hydrochloride, lysine, L-arginine, S-adenosyl-1-methionine, aminobutyric acid, deuterated histamine, heptafluorobutyric acid (HFBA), and methanol LC/MS grade were purchased from Sigma-Aldrich (St. Louis, MO, USA). Water for LCMS was purchased from Fisher Scientific (Fair Lawn, NJ, USA).

#### 2.8.2. LC-HRMS

Liquid chromatography-tandem high-resolution mass spectrometry (LC-HRMS) analysis was performed using a UPLC Ultimate 3000 (Thermo Fisher-Dionex San Jose, CA, USA) system equipped with a HESI-II electrospray source to a Q-Exactive Orbitrap™-based mass spectrometer (all from Thermo Scientific, San Jose, CA, USA). Chromatographic separation was performed on Phenomenex Gemini C18 (100 × 2 mm), 3 µm particle size, the column was held at 37 °C. Peaks were obtained at a flow rate of 0.4 mL min^−1^ with a sample injection volume of 5 µL.

Q-Orbitrap HRMS (Thermo Scientific, San Jose, CA) with HESI-II electrospray source was operated in positive mode. The Xcalibur™ 3.1.66 software (Thermo Scientific, Bremen, Germany) was used to control the instruments and to process the data [36].

#### 2.8.3. Sample Preparation

Protein extraction and precipitation were performed as follows: 250 µL of medium were transferred into an Eppendorf microtube and mixed with 150 µL methanol (containing 0.05% HFBA) and 100 µL water for 50 s. After precipitation, samples were centrifuged for 9 min at 15,000 rpm, and the supernatant was evaporated to dryness at 37 °C under a stream of nitrogen. The residue was reconstituted in 500 µL of methanol/water (20:80 v/v) solution and 50 µL of IS (deuterated histamine). An aliquot of 5 µL of the solution was injected into the HPLC-MS/MS system for analysis.

### 2.9. Statistical Analysis

Statistical analysis was performed using Statistical Package for the Social Sciences version 13 software (SPSS Inc., Chicago, IL, USA). The experiments were performed two times with three technical replicates for each treatment. For this study, Kruskal–Wallis rank sum and Wilcoxon signed-rank test were used, assuming a *p*-value < 0.05 as statistically significant.

## 3. Results

### 3.1. Morphological Analysis of ADSCs Exposed to MCF7-Exhausted Medium

**Figure 2:**
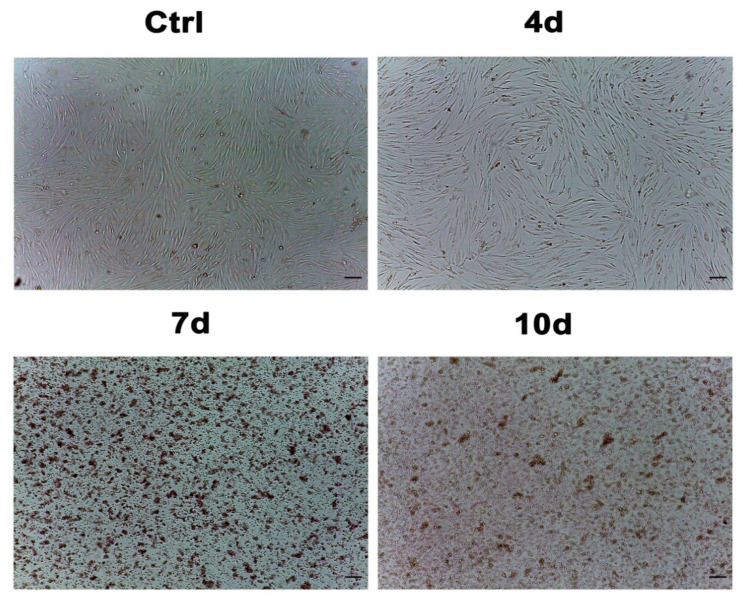
Optical microscope analysis of hADSC morphology after exposure to MCF-7-exhausted media. Figure 2 shows morphological changes in cell treated with 4d-MCF-7-EM (4d), or 7d-MCF-7-EM (7d) or 10d-MCF-7-EM (10d), as compared to untreated control cells (Ctrl). Scale bar = 100 µm.

Figure 2 shows the hADSC morphology evaluated by optical microscopy (Leica, Nussloch, Germany) after 7 days of culturing, according to the above-described conditions. We did not observe significant changes in the morphology of the cells exposed to 4d-MCF-7-EM (4d), while hADSCs that had been exposed to 7d-MCF-7-EM (7d) or 10d-MCF-7-EM (10d) showed significant changes in their morphology, as compared to control untreated cells (Ctrl).

### 3.2. hADSCs Exposed to Exhausted Medium Undergo Cell-Cycle Progression through G1/S Phase

The BrdU assay showed that cell proliferation was increased in hADSCs exposed to 4d-MCF-7-EM (4d - red bars), 7d-MCF-7-EM (7d - green bars), or 10d-MCF-7-EM (10d - blue bars), as compared to control untreated cells (Ctrl – grey bars).

**Figure 3:**
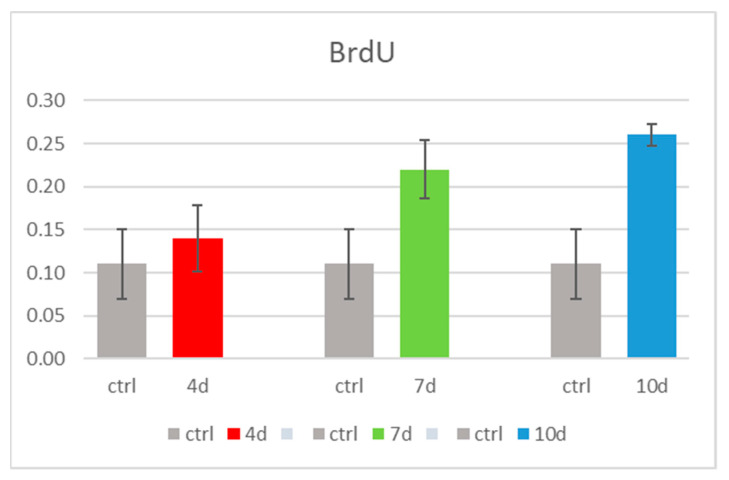
BrdU cell proliferation analysis in hADSCs cultured with 4d-MCF-7-EM (4d - red bars), or 7d-MCF-7-EM (7d - green bars) or 10d-MCF-7-EM (10d - blue bars), as compared to control untreated hADSCs (Ctrl - grey bars).

Cells treated with 10d-MCF-7-EM showed a higher proliferation rate when compared to cells cultured in all the other described conditions, but there is no significant difference compared to control cells (Figure 3).

### 3.3. Exhausted Medium Does Not Affect Cell Viability

We tested different concentrations of the MCF-7 exhausted medium; 100% indicates pure exhausted medium collected after 4, 7, and 10 days of culture; 75% indicates a dilution of 75% of pure exhausted medium collected after 4, 7, and 10 days and 25% growth medium; 50% indicates 50% pure exhausted medium collected after 4, 7, and 10 days, and 50% growth medium; 25% indicates 75% growth medium and 25% pure exhausted medium collected after 4, 7, and 10 days in hADSCs. The control groups were hADSCs cultured in the presence of the growing medium alone.

**Figure 4:**
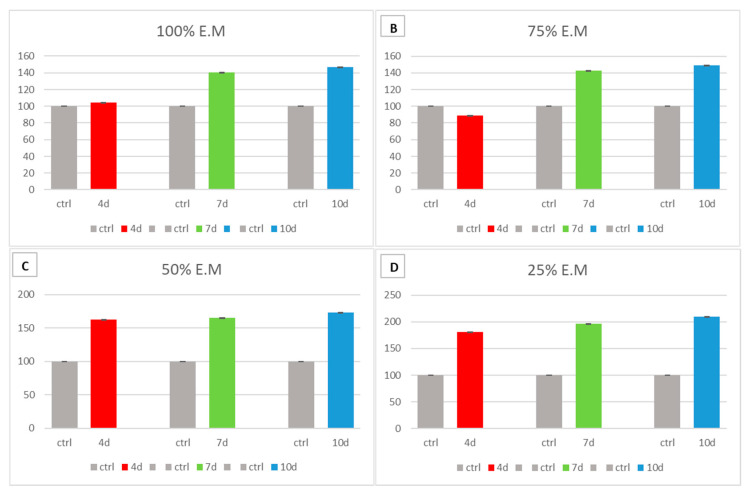
Figure 4 shows the MTT vitality assay. The cytotoxicity of the exhausted medium collected at 4 (red bars), 7 (green bars) and 10 (blue bars) days was evaluated in hADSCs in the presence of different concentrations MCF-7—Exhausted Medium (100% MCF-7—Exhausted Medium (**A**), 75% MCF-7 E.M (**B**), 50% MCF-7 E.M (**C**), and 25% MCF-7 E.M (**D**)). Cell viability was compared to untreated controls hADSCs cultured in the presence of the basic growing medium alone (grey bars) and expressed as absorbance at 570 nm. The experiments were performed with three technical replicates for each treatment. Data are expressed as mean ± SD referred to the control.

MTT assay shows that pure exhausted medium and dilutions did not reduce hADSC viability as compared to untreated control cells (Figure 4). Moreover, the pure media (100%) was the only one able to guarantee a constant release of its components for each observed time point (Supplementary Material).

### 3.4. Exposure of hADSCs to Exhausted MCF- 7 Medium Increases Stem Cell Potency

**Figure 5:**
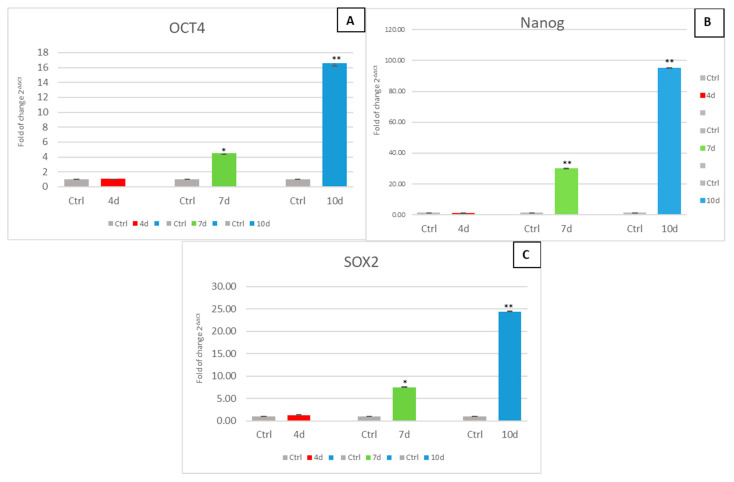
Expression of stemness genes. The expression of stemness-related genes OCT4 (Panel (**A**)), Nanog (Panel (**B**)), and SOX2 (Panel (**C**)) was evaluated in hADSCs cultured with 4d-MCF-7-EM (4d – red bars), 7d-MCF-7-EM (7d – green bars), or 10d-MCF-7-EM (10d – blue bars). The mRNA levels for each gene were normalized to Glyceraldehyde-3-Phosphate-Dehydrogenase (GAPDH) and expressed as fold of change (2^−ΔΔCt^) of the mRNA levels observed in untreated control hADSCs (Ctrl - grey bars) defined as 1 (mean ± SD; *n* = 6). Data are expressed as mean ± SD referred to the control (* *p* ≤ 0.05), (** *p* ≤ 0.01).

Figure 5 shows the mRNA levels of stemness-related genes in hADSCs exposed to 4d-MCF-7-EM (4d-red bars), 7d-MCF-7-EM (7d-green bars) or 10d-MCF-7-EM (10d-blue bars) in culture. The expression of all the tested stemness genes was significantly upregulated in cells cultured in the presence of the exhausted medium, as compared to control untreated cells (Ctrl - grey bars).

### 3.5. Exhausted MCF- 7 Medium Influence on p53, p21 (WAF1/CIP1) and BAX

**Figure 6:**
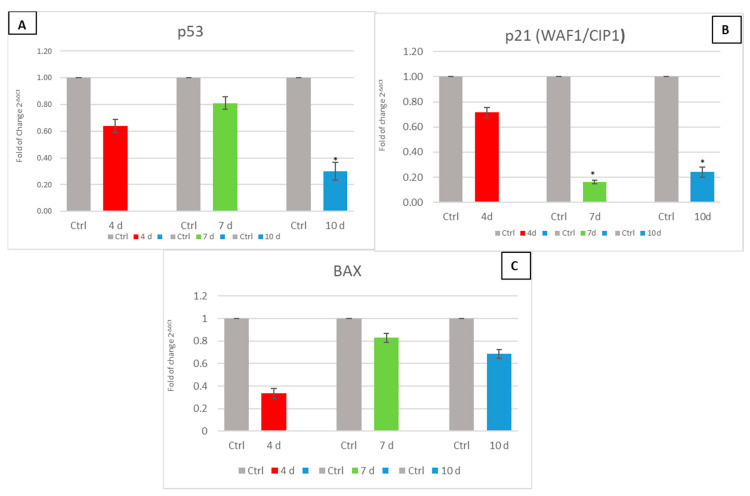
The expression of p53 (Panel (**A**)), p21 (WAF1/CIP1) (Panel (**B**)), and BAX (Panel (**C**)) was evaluated in human Adipose-derived stem cells (hADSCs) cultured with 4d-MCF-7-EM (4d – red bars), 7d-MCF-7-EM (7d – green bars), or 10d-MCF-7-EM (10d – blue bars). The mRNA levels for each gene were normalized to Glyceraldehyde-3-Phosphate-Dehydrogenase (GAPDH) and expressed as fold of change (2^−ΔΔCt^) of the mRNA levels observed in untreated control hADSCs (Ctrl - Grey bar) defined as 1 (mean ± SD; *n* = 6). Data are expressed as mean ± SD referred to the control (* *p* ≤ 0.05).

Figure 6 shows the mRNA levels of cell cycle-related genes p53 and p21 (WAF1/CIP1) and pro-apoptosis gene BAX in hADSCs exposed to 4d-MCF-7-EM (4d-red bars), 7d-MCF-7-EM (7d-green bars), or 10d-MCF-7-EM (10d-blue bars). In medium-treated cells, the expression of the tested genes was downregulated, as compared to untreated controls (Ctrl-grey bars).

### 3.6. Exhausted MCF- 7 Medium Affects Epigenetic Modulating Genes in hADSCs

**Figure 7:**
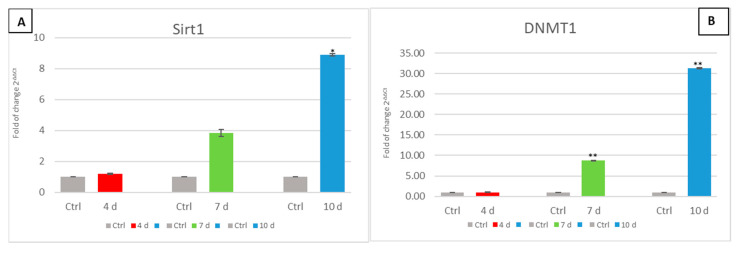
Expression of epigenetic modulating genes. The expression of SIRT1 (Panel (**A**)) and DNMT1 (Panel (**B**)) was evaluated in hADSCs cultured in the presence of 4d-MCF-7-EM (4d - red bars), 7d-MCF-7-EM (7d - green bars), or 10d-MCF-7-EM (10d - blue bars). The mRNA levels for each gene were normalized to Glyceraldehyde-3-Phosphate-Dehydrogenase (GAPDH) and expressed as fold of change (2^−ΔΔCt^) of the mRNA levels observed in untreated control hADSCs (Ctrl - grey bar) defined as 1 (mean ± SD; *n* = 6). Data are expressed as mean ± SD referred to the control (* *p* ≤ 0.05), (** *p* ≤ 0.01).

The gene expression of SIRT1 and DNMT1 (Figure 7) was investigated in hADSCs cultured in the presence of an MCF-7 exhausted medium. The expression of these epigenetic genes was significantly upregulated only in ADSCs that had been exposed to 7d-MCF-7-EM (7d-green bars), or 10d-MCF-7-EM (10d-blue bars), as compared to control untreated cells (Ctrl-grey bars).

### 3.7. MCF7-Exhausted Media Induce Autophagy in hADSCS

**Figure 8:**
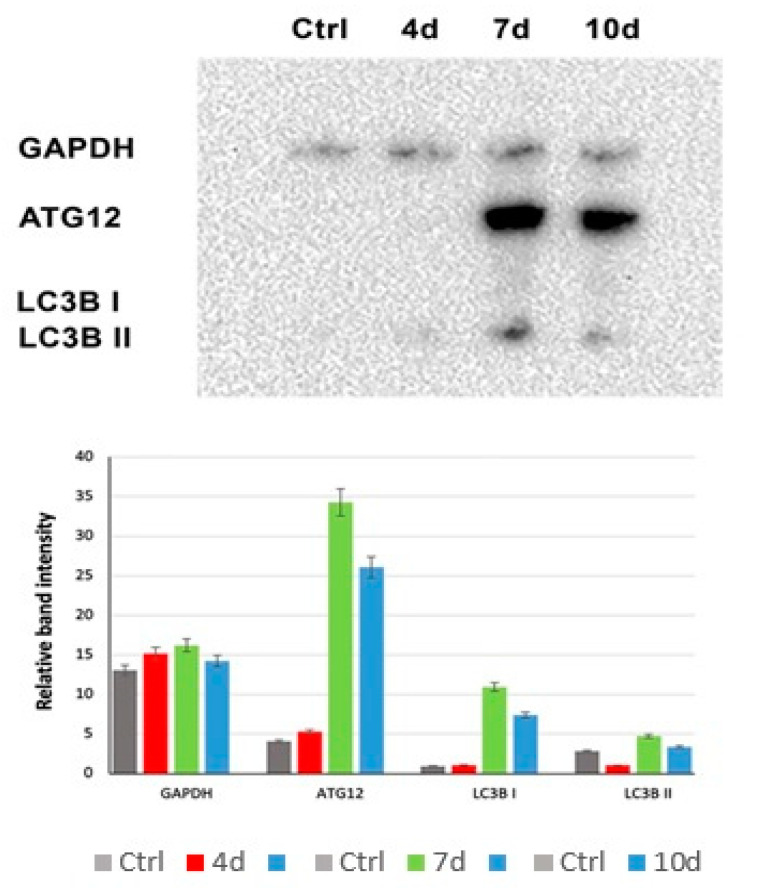
Analysis of autophagosome formation. The protein levels were analyzed on hADSCs cultured with 4d-MCF-7-EM (4d - red bars), 7d-MCF-7-EM (7d - green bars), or 10d-MCF-7-EM (10d - blue bars) and control cells (Ctrl - grey bars) after 7 days by Western blot, using an autophagosome marker antibody sampler kit. The sizes of the bands were determined using pre-stained marker proteins. The data presented are representative of different independent experiments.

Western blotting analysis of autophagosome formation (Figure 8) showed the activation of autophagy in hADSCs exposed to 7d-MCF-7-EM (7d–green bars) or 10d-MCF-7-EM (10d–blue bars), exhibiting higher expression levels of LC3B I and LC3B II and Atg12 proteins, as compared to control untreated cells (Ctrl–grey bars) and to 4d-MCF-7-EM (4d–red bars).

### 3.8. Polyamine Detection

The polyamine content was evaluated in 4d-MCF-7-EM, 7d-MCF-7-EM, or 10d-MCF-7-EM and compared to the control represented by MCF-7 medium alone (referred to as Ctrl). The results in the table show a significant difference in the content of polyamines as compared to the controls (*** *p* ≤ 0.001) for all the analyzed molecules (Table 2).

## 4. Discussion

Breast reconstruction after mastectomy has become an integral part of breast cancer treatment, giving women the chance to alleviate some of the emotional and aesthetic psychological effects of this disease [40,41]. Adipose tissue represents a valuable source of mesenchymal stem cells [42], easily collectible, able to differentiate toward different cell lineages upon stimulation, therefore representing a suitable tool in regenerative and aesthetic medicine or plastic surgery procedures, such as breast reconstruction after mastectomy [43].

Nevertheless, some issues are still open. In particular, the clear evidence that stem cell behavior and fate are influenced by the cellular microenvironment [5,6,7,10,37] raises more than a cautionary note on the use of a stem cell source in patients who underwent mastectomy as a part of a breast cancer treatment.

In this study, we evaluated whether a conditioned medium harvested at different time points from breast cancer cells may have been acting on hADSCs to affect their pluripotency, epigenetic gene expression, and autophagy.

The MCF-7 cells represent a model cell line suitable for important research investigations and experiments on breast cancer cells, including anticancer drugs [44]. They release in the medium several factors, such as cytokines, exosomes, and polyamines (Table 2) [45,46] that can induce changes in gene expression (Figure 5, Figure 6 and Figure 7) and morphology (Figure 2). Other authors previously demonstrated the ability of this medium to induce a transformation in hADSCs toward cancer-associated fibroblasts (CAF) [47,48]. Although transformed, they retain some typical markers of mesenchymal stem cells [49].

Our results show that the expression of stemness genes (OCT4, Sox2, Nanog) was significantly upregulated in hADSCs that had been cultured in the presence of MCF-7-EM and that the exposure effect occurred in a time-dependent fashion.

It is worth considering that the overexpression of these genes could be associated with the initiation of an uncontrolled transformation leading to a malignant phenotype, as it has been documented elsewhere [37].

The analysis of the mRNA levels of p53 and p21 (WAF1/CIP1), involved in cell-cycle arrest, revealed the capability of the MCF-7-EM to downregulate the expression of both genes in hADSCs, without altering their viability. Moreover, p53 is associated with the activation of apoptosis through the activation of pro-apoptotic proteins such as BAX [50]. From our results, MCF-7-EM exposure elicited a decrease in the hADSC gene expression of both p53 and BAX, thus indicating the generation of an anti-apoptotic, pro-proliferative state.

Such an indication is further inferred from the observation that SIRT 1 was overexpressed following hADSC exposure to MCF-7-EM. In actual fact, SIRT1 is known to inhibit the expression of p53-regulated genes, inducing p53 deacetylation, thus preventing cellular arrest and apoptosis. Interestingly, not only p53 but even p21p21 (WAF1/CIP1) gene expression was inhibited in MCF-7-EM-exposed hADSCs. p53 activates transcription of p21 (WAF1/CIP1), a cell-cycle inhibitor that promotes both cdk/cyclin inhibition and cell-cycle arrest during the G1/S phase [24,51,52]. The currently observed inhibition in p21 gene expression further corroborates the environmental pressure exerted by MCF-7-EM toward a de-regulated proliferative state executed within a risky context of stemness enhancement.

Consonant with this view is the observed increase in DNMT1 transcription in MCF-7-EM treated hADSCs. Such overexpression could be related to the maintenance of a methylated state in genes responsible for differentiation, such as RUNX 2 and PPAR γ, which are conversely hypomethylated in hADSCs undergoing differentiation [53]. This result is particularly interesting within our observed context of MCF-7-EM-induced increase in stemness gene expression since DNMT1 is induced by OCT4 and Nanog by direct binding to its promoter [54].

In order to identify a potential group of molecules capable of orchestrating the molecular changes observed in MCF-7-EM treated hADSCs, we decided to investigate the presence of different polyamines in the MCF-7-EM, considering the ability of these molecules to regulate multifaceted, often intertwined cellular processes. It has been shown that the downregulation of some polyamines belonging to the arginine pathway like spermidine causes an arrest in the cell cycle, inhibiting cell proliferation [55].

Compounding the pro-stemness/pro-proliferative, anti-apoptotic environment provided by stem cell exposure to MCF-7-EM, we found that this medium, collected after 4, 7, or 10 days of culturing, expressed significantly different levels of polyamines, as compared with the control medium alone.

The intracellular metabolism of polyamines can be considered a cyclic process, allowing the transformation of a polyamine into another, finely tuning both their accumulation and depletion [56]. The formation of polyamines occurs at the cytoplasmic level, where putrescine, through the action of S-adenosyl-methionine, generates spermidine, which, in turn, by the aid of a transferase, leads to the formation of spermine [57].

Spermine can be converted back into spermidine and spermidine into putrescine via acetylation. These molecules can be released from the cell into the extracellular environment, acting both in a paracrine and autocrine fashion [58,59].

Here, we show higher levels of spermidine and acetylated spermine in MCF-7-EM, as compared to the control medium alone, indicating that these two molecules are rapidly produced by MCF-7. Spermidine plays a key role in cell-cycle progression, as shown by the observation that its rapid depletion caused a total inhibition of protein synthesis and growth arrest [60]. Conversely, both spermidine and acetylated polyamines have been shown to be involved in cell proliferation [61,62].

On these bases, we can hypothesize a role of these molecules, released from MCF-7, as intercellular messengers modulating hADSCs proliferation.

The hypothesis that spermidine produced by MCF-7 could be involved in hADSC transformation contributing to their higher proliferation rate is also in agreement with the downregulation in p53 and p21 (WAF1/CIP1) expression observed here following stem cell exposure to MCF-7-EM since the cell-cycle arrest induced by polyamine depletion is known to be associated with the induction of these cell-cycle inhibitors [55]

Moreover, spermidine can induce autophagy [63]. Autophagy has been shown to be able to maintain stemness, playing a role in stem cell differentiation toward specific lineages, by degrading unwanted organelles and proteins while simultaneously providing building blocks for the neosynthesized biomolecules [64]. Autophagy can be initiated in response to nutrient deprivation and various other stresses [65].

In actual fact, we investigated whether in hADSCs cultured in the presence of MCF-7-EM, a different expression of autophagy could be observed. For this purpose, we analyzed the main proteins involved in autophagosome formation, Atg12, LC3B I, and Lc3B II (Figure 8) [66], observing higher levels of all these proteins in hADSCs that had been exposed to 7d-MCF-7-EM and in 10d-MCF-7-EM. The autophagy induced by MCF-7-EM in hADSCs is consonant with the higher proliferation rate observed since autophagy was recently associated with stemness maintenance and inhibition of differentiation [64].

Nevertheless, the levels of spermine, putrescine, and acetyl-spermidine were significantly reduced in MCF-7-EM when compared to the control medium. Additional studies will be required to dissect the effects elicited on hADSC proliferation by the timely changes in the differential expression of these molecules within MCF-7-EM.

## 5. Conclusions

The present observations indicate that factors contained in the exhausted medium of a breast cancer cell line are able to remarkably influence stem cell behavior in vitro by eliciting an increased expression of stemness-related genes and epigenetic genes, together with an increase in cell proliferation, a blockade of differentiation, and maintenance of stem cell potency.

These findings suggest that hADSCs, which are currently transplanted in an increasing number of diseased tissues, may drift to a deregulated and dangerous highly proliferative phenotype when transplanted in a pathological environment in vivo.

## Figures and Tables

**Table 1:** Primer sequences.

Primer Name	Forward	Reverse
OCT4	GAGGAGTCCCAGGCAATCAA	CATCGGCCTGTGTATATCCC
SOX2	CCGTTCATGTAGGTCTCGGAGCTG	CAACGGCAGCTACAGCTAGATGC
Nanog	CATGAGTGTGGATCCAGCT	CCTGAATAAGCAGATCCAT
SIRT1	CATTTCCATGGCGCTGAGG	TGCTGGTGGAACAATTCCTGT
DNMT1	CGTCCGAGCGTCACACA	GAGCCTTTGCCATTCTTCGC
p53	CAAGCAATGGATGATTTGATGCT	TGGGTCTTCAGTGAACCATTGT
p21 (WAF1/CIP)	CAAAGGCCCGCTCTACATCTT	AGGAACCTCCATTCACCCGA
BAX	TGCTTCAGGGTTTCATCCAG	GGCGGCAATCATCCTCTG
GAPDH	GAGTCAACGGATTTGGTCGT	GACAAGCTTCCCGTTCTCAG

**Table 2:** Amounts of polyamines (ng/mL) in 4d-MCF-7-EM, 7d-MCF-7-EM, or 10d-MCF-7-EM and MCF-7 fresh medium (Ctrl). The *p*-value is related to the change in concentration of the analytes listed in the table in the medium analyzed.

Polyamines	Ctrl	4d-MCF-7-EM	7d-MCF-7-EM	10d-MCF-7-EM	*p*-Value
Putrescine	0.79	0.68	0.63	0.60	0.0005 ***
Spermidine	0.98	1.054	1.27	1.35	0.0009 ***
Spermine	1.29	1.22	1.09	0.86	0.001 ***
Agmantine	0.8	0.65	0.60	0.61	0.0007 ***
N-acetylputrescine	0.38	0.38	0.42	0.42	0.00003 ***
N-acetylspermine	0.411	0.420	0.510	0.515	0.0005 ***
N-acetylspermidine	0.322	0.284	0.268	0.270	0.0002 ***

Amounts of polyamines (ng/mL). Data are expressed as mean ± SD referred to the control (*** *p* ≤ 0.001).

## Data Availability

Not applicable.

## Supplementary Materials

The following are available online at https://www.mdpi.com/article/10.3390/cells10071754/s1, Figure S1: Representative chromatograms of analytes in HRMS Orbitrap, Table S1: Calibration curves, Figure S2: Calibration curve and medium release.
