# Supplementary material for: Adipose-Derived Stem Cell Features and MCF-7
Source: Cells. 2021 Jul 11;10(7):1754. doi: 10.3390/cells10071754 (PMC8307920; doi:10.3390/cells10071754)
Supplement: Supplementary file 1 [file cells-10-01754-s001.zip › cells-1273087-supplementary.pdf]

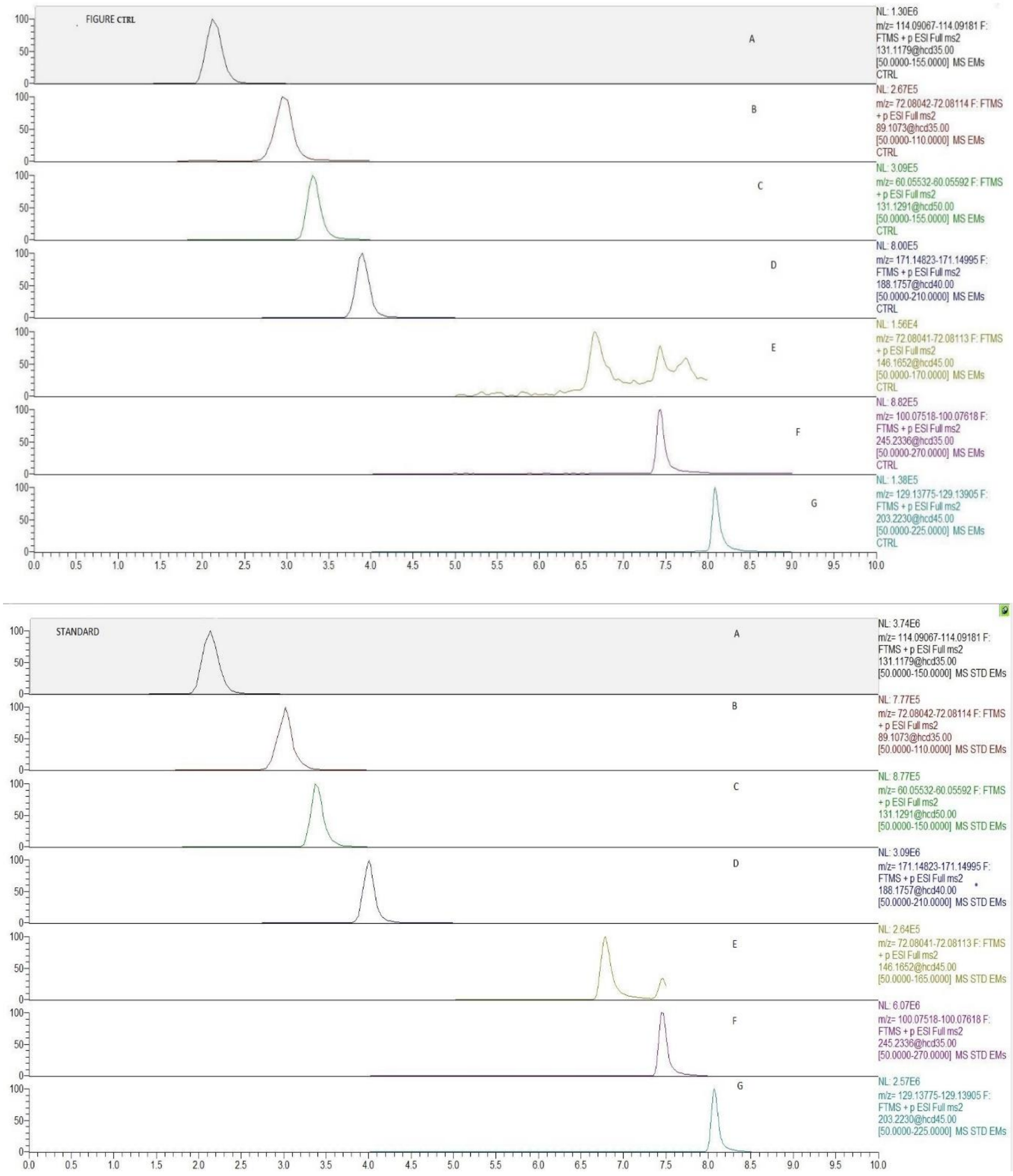

Figure S1. Representative chromatograms of analytes in HRMS Orbitrap with PRM mode in MCF-7 medium (indicated as CTRL) and in MCF-7 EM (indicated as Standard): (A) N-Acetyl-putrescine, (B) Putrescine, (C) Agmatine, (D) N(1)-Acetyl-spermidine, (E) Spermidine, (F) N1-Acetylspermine, (G) Spermine.

Table S1. Calibration curves were prepared for each analyte, which were linear, and the calibration regression coefficients ranged from 0.99 to 0.9965 for all analytes:

ANALYTE	FORMULA	EXPERIMENTHAL MASS (*m/z*)	THEORETICAL MASS (*m/z*)	FRAGMENT MASS	R^2
PUTRESCINE	$C_4H_{12}N_2$	89.10743	89.10732	72.08128	0.9991
SPERMIDINE	$C_7H_{19}N_3$	146.16505	146.16517	72.08077	0.9925
SPERMINE	$C_{10}H_{26}N_4$	203.22232	203.22302	129.13844	0.9900
AGMATINE	C5H14N4	131.12906	131.12912	114.10258	0.9965
N-ACETYLPUTRESCINE	$C_6H_{14}N_2O$	131.11783	131.11789	114.09124	0.9960
N-ACETYLSPERMINE	$C_{12}H_{28}N_4O$	245.23291	245.23359	100.07568	0.9934
N-ACETYLSPERMIDINE	$C_9H_{21}N_3O$	188.1756	188.17574	171.14909	0.9947

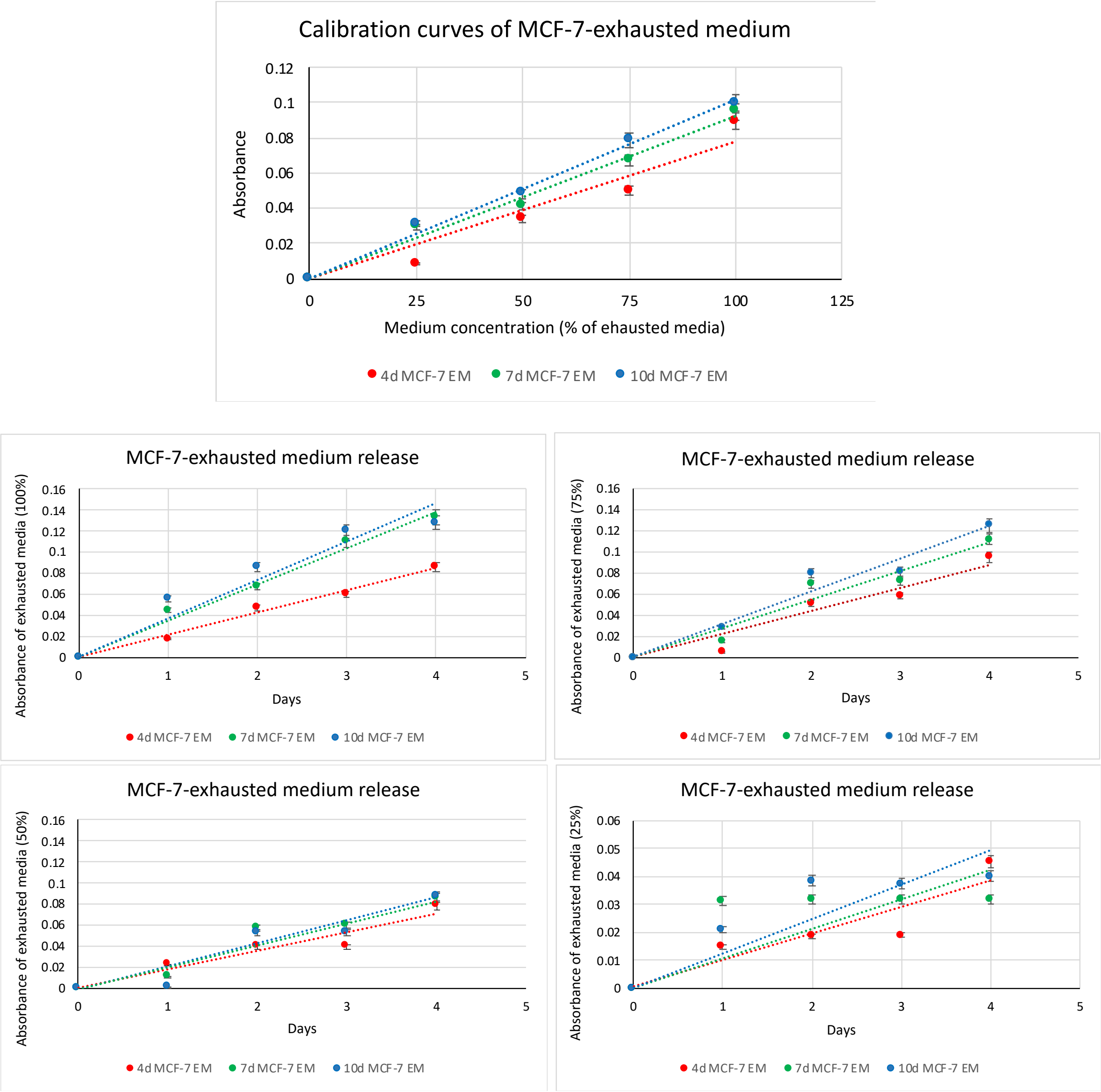

Figure S2. Calibration curve and medium release. MCF-7 cell culture medium was composed of Dulbecco's modified Eagle's Medium (DMEM), (Life Technologies Grand Island, NY, USA) supplemented with 10% fetal bovine serum (FBS) (Life Technologies, Grand Island, NY, USA), 200 mM L-glutamine (Euroclone, Italy), and 200 U/mL penicillin—0.1 mg/mL streptomycin (Euroclone, Milano, Italy). The exhausted medium was collected from MCF-7, after 4 (4d-MCF-7-EM), 7 (7d-MCF-7-EM), and 10 (10d-MCF-7-EM) days in culture. Calibration curve was prepared by spectrophotometric absorbance readings of scalar dilutions of the MCF-7-exhausted medium (0, 25%, 50%, 75% and 100%). We then monitored over time the variation of absorbance of the various media and observed that only the pure media (100%), was the only one able to guarantee a constant release of its components for each observed time points.
